# Evaluation of In Vitro Genotoxicity of Polystyrene Nanoparticles in Human Peripheral Blood Mononuclear Cells

**DOI:** 10.3390/toxics11070627

**Published:** 2023-07-20

**Authors:** Milda Babonaitė, Matas Čepulis, Jūratė Kazlauskaitė, Juozas Rimantas Lazutka

**Affiliations:** Department of Botany and Genetics, Institute of Biosciences, Vilnius University, 7 Sauletekis Av., LT-10257 Vilnius, Lithuania; cepulis.mat@gmail.lt (M.Č.); jurate.mierauskiene@gf.vu.lt (J.K.); juozas.lazutka@gf.vu.lt (J.R.L.)

**Keywords:** nanoplastic, polystyrene nanoparticles, peripheral blood lymphocytes, comet assay, micronucleus assay

## Abstract

According to the trade association PlasticEurope, global plastics production increased to 390.7 million tons in 2021. Unfortunately, the majority of produced plastics eventually end up as waste in the ocean or on land. Since synthetic plastics are not fully biodegradable, they tend to persist in natural environments and transform into micro- and nanoplastic particles due to fragmentation. The presence of nanoplastics in air, water, and food causes ecotoxicological issues and leads to human exposure. One of the main concerns is their genotoxic potential. Therefore, this study aimed to evaluate the internalization rates, cytotoxicity, and genotoxicity of polystyrene nanoparticles (PS-NPs) in human peripheral blood mononuclear cells (PBMCs) in vitro. The uptake of PS-NPs was confirmed with flow cytometry light scattering analysis. None of the tested nanoparticle concentrations had a cytotoxic effect on human PBMCs, as evaluated by a dual ethidium bromide/acridine orange staining technique. However, an alkaline comet assay results revealed a significant increase in the levels of primary DNA damage after 24 h of exposure to PS-NPs in a dose-dependent manner. Moreover, all tested PS-NPs concentrations induced a significant amount of micronucleated cells, as well. The results of this study revealed the genotoxic potential of commercially manufactured polystyrene nanoparticles and highlighted the need for more studies with naturally occurring plastic NPs.

## 1. Introduction

Over the last few decades, world plastic production has grown significantly. According to the trade association PlasticEurope, global plastics production increased to 390.7 million tons in 2021 alone, where 90.2% of the production was fossil-based, and just 9.8% was post-consumer recycled plastics and bio-based plastics [1]. Unfortunately, the majority of produced plastics eventually end up as waste in the ocean or on land. Since synthetic plastics are largely not fully biodegradable, they tend to persist in natural environments and, due to fragmentation, transform into smaller micro- and nanoplastic particles [2,3,4]. As suggested by Gigault et al. [5], nanoplastics are particles with a size range of 1 to 1000 nm that are produced by the breakdown of industrial plastic products and might display colloidal properties. Currently, plastics can be composed of a wide range of polymers, including polypropylene (PP), polyethylene terephthalate (PET), polystyrene (PS), polyvinyl chloride (PVC), and others [5]. In the past few years, the term “nanoplastic” has drawn a lot of interest and concern due to possible human exposure. In 2019, the World Health Organization reported that plastic particles can be found in tap and bottled water [6]. In 2022, scientists discovered different nano-sized plastic particles in two of the world’s most remote places: central Greenland and Antarctica [7]. Micro- and nanoplastics can already be detected in a variety of foods, including aquatic products, fruits and vegetables, and even meat and milk [8,9,10,11]. Thus, the presence of nanoplastics in the air, water, and food not only causes ecotoxicological issues but results in human exposure as well [5,12,13]. Most likely, plastic nanoparticles enter the human body via ingestion or inhalation [3]. In recent studies by Salvia et al. [14] and Leslie et al. [15], the existence of plastic particles in human peripheral blood was confirmed. In the latter study [15], plastic particles were found in almost 80% of the people tested. Furthermore, it was shown that polystyrene nanoparticles could even penetrate the blood–brain barrier [16]. It is thought that plastic particles enter the bloodstream either via mucosal contact or are carried by specific cell types, yet the exact pathway remains undetermined [15].

One of the most predominant types of plastic nanoparticles in the environment is polystyrene nanoparticles (PS-NPs) [3]. Recent studies revealed that there are still some inconsistencies in the evaluation of the cytotoxic and genotoxic potential of PS-NPs. In some studies, PS-NPs were not able to induce any DNA damage in human intestinal epithelial Caco-2 cells [17] and other human cell models [18], while others suggest that PS-NPs are competent to induce primary DNA damage, intracellular ROS production, and chromosomal damage [19,20,21]. Variations in the results are thought to be due to different sizes and concentrations of PS-NPs used as well as the specificities of different cell lines.

In the past few years, studies on PS-NPs genotoxicity in more realistic human-like models, such as human peripheral blood mononuclear cells (PBMC), were carried out and confirmed the genotoxic potential of PS-NPs [22,23,24,25]. A statistically significant increase in the frequency of micronuclei and chromosome aberrations was reported in the study of Sarma et al. [22], where peripheral blood lymphocytes (PBLs) were exposed to 50 nm size PS-NPs concentrations from 500 to 2000 µg/mL. These findings suggest that 50 nm PS-NPs have the potential to induce chromosomal damage and genomic instability in lymphocyte cells. In addition, the genotoxicity and immunomodulatory effect studies of PS-NPs were conducted by Ballesteros et al. [21], confirming the uptake of PS-NPs by white blood cells and showing significantly increased levels of DNA damage in monocytes and polymorphonuclear cells treated with 100 µg/mL PS-NPs concentration [23]. Moreover, an investigation with differently sized PS-NPs reported stating that PS-NPs increased ROS levels and induced lipid and protein oxidation [24]. Following the latter study [24], the investigation of the genotoxic action of the same PS-NPs was conducted. It was reported that PS-NPs increased single/double-strand break formation, oxidized purines and pyrimidines levels, and 8-oxo-2′-deoxyguanosine formation [25].

Although the number of studies involving PS-NP cyto-genotoxicity in peripheral blood lymphocytes is increasing, in a majority of studies, genotoxicity is evaluated only at one endpoint—either primary or oxidative DNA damage or chromosomal damage. Therefore, the aim of this study was to evaluate the cytotoxic and genotoxic effects of polydisperse (0.05 nm–0.1 µm) polystyrene nanoparticle suspension in human peripheral blood mononuclear cells and explore potential risks that might be associated with human exposure to nanoplastics. Cytotoxicity was assessed by dual ethidium bromide/acridine orange (EB/AO) staining technique. To determine the genotoxic effects of PS-NPs, alkaline comet assay and micronucleus assay were applied. These two methods are often used in combination due to their complementarity to each other, where alkali comet assay detects general DNA damage, such as DNA strand breaks and alkali-labile sites, and MN assay can evaluate the clastogenic and aneugenic effects [26].

## 2. Materials and Methods

### 2.1. Particle Characterization and Preparation of NPs Suspensions

PS-NPs with a size ranging from 0.05 to 0.1 µm (PS-NPs, PP-008-10) were purchased from Spherotech (Chicago, IL, USA). Dispersions of PS-NPs were prepared at 1 mg/mL (0.1%) in distilled water and in the cell culture medium (RPMI, 10% fetal bovine serum) and sonicated at 35 kHz for 30 min in Bandelin Sonorex Super sonication bath (BANDELIN electronic GmbH & Co. KG, Berlin, Germany). Hydrodynamic particle size was analyzed by Nanoparticle Tracking Analysis (NTA) (NanoSight LM10, Malvern Panalytical Ltd., Malvern, UK) immediately (0 h), 3 h, and 24 h after sonication. The samples were injected into the chamber with sterile syringes until the liquid reached the tip of the nozzle. Each measurement was performed at 22 °C. The NanoSight NTA 3.1 analytical software was employed, and the viscosity of water was used as the input value.

The 1 mg/mL (0.1%) and 5 mg/mL (0.5%) PS-NPs solutions in the RPMI medium were prepared for cyto-genotoxicity studies. NPs suspensions were sonicated at 35 kHz for 30 min and immediately used. For cytotoxicity evaluation and comet assay, cells were treated with 0.1% PS-NPs solutions (final concentrations ranging from 15 to 100 µg/mL), whereas for micronucleus assay, 0.5% suspension was used (final concentrations ranging from 25 to 125 µg/mL). Even though the concentrations of naturally occurring plastic nanoparticles that humans are exposed to are lower than those tested in this study, the natural exposure is generally thought to be chronic [27]. On the contrary, in this study, we evaluated an acute effect of PS-NPs, explaining the selected higher concentrations, which partly coincide with the doses chosen by other researchers [20,22,23].

### 2.2. Biological Material

The study was conducted on human peripheral blood in vitro. Human peripheral blood was selected because it represents a heterogeneous population of cells and is OECD-validated. Peripheral blood samples from five healthy 22–29-year-old volunteer donors (non-smoking with no known illness) were collected by venipuncture in heparinized vacutainer tubes (Becton-Dickinson, Franklin Lakes, NJ, USA). Whole blood samples from three donors were used for micronucleus assay, whereas isolated mononuclear cells from five donors were used for cytotoxicity analysis and comet assay. Informed consent was obtained from all subjects involved in the study. The study was conducted in compliance with research ethics requirements adopted by Vilnius University.

### 2.3. Lymphocyte Isolation

Isolation of PBMCs was performed with density gradient centrifugation medium Fiqoll-Paque^TM^, according to the manufacturer‘s instructions. Briefly, equal parts of the blood and RPMI cell medium were added to a conical tube, and an equal part of Ficoll-Paque^TM^ was slowly placed at the bottom using a Pasteur pipette. The resulting sample was centrifuged at room temperature at 800× *g* for 20 min to isolate cells according to their cellular density. Then, the mononuclear cell layer was carefully aspirated, transferred into a new tube, washed with RPMI cell medium, and centrifuged again at 800× *g* for 10 min at room temperature. Finally, the supernatant was discarded, and the pellet was resuspended at 1 × 10^5^ cells/mL in the RPMI cell medium for comet assay and at 1 × 10^6^ cells/mL for cytotoxicity evaluation.

### 2.4. NPs Uptake Analysis

The uptake potential of NPs in PBMCs was assessed using a flow cytometry light scatter analysis, according to Suzuki et al. [28]. Following PBMCs isolation, cells were resuspended in RPMI 1640 medium at a concentration of 4 × 10^5^, transferred into sterile 15 mL tubes, and treated with several concentrations (0–500 µg/mL) of PS-NPs for 24 h. After exposure to NPs, the cells were centrifuged at 800× *g* for 10 min, the supernatant was removed, and the cells were resuspended in phosphate-buffered saline (PBS). The levels of particles taken up by cells were evaluated using a flow cytometer using a 488 nm laser beam. A total of ten thousand cells were analyzed for each sample. Data processing was carried out through Floreada.io software. The intensities of forward-scatter(ed) (FSC) light, which represent the size of cells, and side-scatter(ed) light, which is proportional to the intracellular density and granularity, which reflects the NPs uptake, were measured [28].

### 2.5. Analysis of Cell Viability and Primary DNA Damage (the Comet Assay)

Once the uptake of PS-NPs by PBMCs was confirmed, the cytotoxicity and genotoxicity of these particles were evaluated in both time- and concentration-dependent manner by dual AO/EB staining and alkaline comet assay, respectively. Peripheral blood mononuclear cells of five donors were treated with PS-NPs in concentrations ranging from 15 to 100 µg/mL for 3 and 24 h at 37 °C in a 5% CO_2_. As a positive control, 20 µM hydrogen peroxide was added 1 h prior to the end of the incubation time. Untreated control (0 µg/mL) served as a reference point, representing the highest level of viability and the lowest level of primary DNA damage. Exposure was conducted in sterile 15 mL centrifuge tubes. After incubation, samples were centrifuged at 800× *g* for 10 min. The supernatant was discarded, and the pellet was resuspended in 1 mL of RPMI 1640 cell culture medium.

For cytotoxicity evaluation, dual acridine orange-ethidium bromide (AO/EB) staining was used, according to Liu et al. [29], with some modifications. Briefly, AO/EB dye mix was prepared by combining 1 µL of ethidium bromide (EB) (3 mg/mL) and 1 µL of acridine orange (AO) (5 mg/mL) with 1 mL PBS. Finally, 1 µL of resulting AO/EB stain was mixed with 10 µL of cell suspension, placed onto a clean microscope slide, covered with a cover slip, and examined under 20× objective with a fluorescent microscope and filter combination suitable for reading fluorescein. AO permeates all cells, intercalates into double-stranded DNA, and, upon 488 nm excitation, emits a green fluorescence [30]. EB is only taken up by dead or late apoptotic cells when cytoplasmic membrane integrity is disrupted and stains the nucleus red. In addition, EB dominates over AO. Therefore, live cells have a green nucleus, and late apoptotic or dead cells have a bright orange or red nucleus, respectively [31]. At least 100 cells of each sample were scored to determine the percentage of green (viable) and orange/red (dead) cells. The formula used to establish viability was [32]

(1) $$\%\;viable\;cells=\frac{number\;of\;viable\;cells}{total\;number\;of\;cells}\times 100$$

The primary DNA damage was determined by alkaline comet assay, according to Singh et al. [33], and comet assay procedures described according to MIRCA [34]. Forty µL of cell suspension was mixed with forty µL of fresh 1% low melting point (LMP) agarose in PBS, pH 7.4, at 37 °C (final agarose concentration 0.5%). Then, 80 µL of cell and agarose mixture was pipetted onto frosted glass microscope slides, precoated with 1% normal melting point (NMP) agarose, and covered with a coverslip (24 × 24 mm). Two sets of duplicate gels per sample were prepared. The agarose was left to solidify for 10 min at 4 °C, coverslips were removed, and slides were immersed in cold, freshly prepared lysis solution (2.5 M NaCl, 100 mM Na_2_EDTA, 10 mM Tris, with 1% Triton X-100 and 10% DMSO added just before use, pH 10) at 4 °C for 90 min to remove cellular proteins. After that, slides were placed in a cooled to 4 °C horizontal gel electrophoresis tank COMET—20 SYSTEM (Scie-Plas, Cambridge, UK) filled with cold fresh electrophoresis buffer (1 mM Na_2_EDTA and 300 mM NaOH, pH 13) and left for 20 min to facilitate DNA unwinding. Then, electrophoresis was carried out at 19 V and 300 mA (1.1 V/cm) for 30 min. To keep the temperature of the buffer constant, it was additionally circulated and cooled. Following electrophoresis, slides were washed with a cold neutralization buffer (0.4 M Tris HCl, pH 7.5) for 30 min at 4 °C. Finally, samples were stained with 80 µL of ethidium bromide (20 µg/mL). All the above steps were conducted under dimmed light to prevent additional DNA damage.

**Figure 1:**
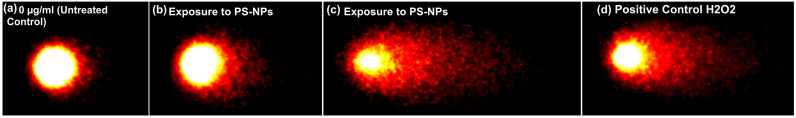
Representative fluorescent images of stained comets using human PBMCs. (**a**) Untreated/negative control, (**b**,**c**) cells exposed to PS-NPs, (**d**) H_2_O_2_ (as a positive control). Images were captured using a 40× objective.

The slides were examined by a single scorer using a fluorescence microscope (Nikon Eclipse 80i, Nikon, Tokyo, Japan) at 400× magnifications. Image capture and analysis were performed using LUCIA Comet Assay™ software (Laboratory Imaging, s.r.o., Prague, Czech Republic). For each sample, 2 gels were prepared, and 50 randomly chosen nucleoids per gel were scored (a total of 100 comets per sample). The comet’s head represents intact DNA, while the tail represents fragmented DNA [35] (Figure 1). The percentage of DNA in the comet tail (% TDNA) was used as a parameter of DNA damage. The overall level of DNA migration was expressed as the mean value ± SEM.

### 2.6. The Micronucleus Assay

Chromosomal damage was determined using the micronucleus assay that was performed according to Fenech [36]. Heparinized whole blood from three healthy donors was diluted in the ratio of 1:15 with HEPES-buffered RPMI 1640 medium supplemented with 12% heat-inactivated newborn calf serum, 7.8 μg/mL phytohemagglutinin P (PHA), and 50 μg/mL gentamycin. Cell cultures were incubated in glass vials at 37 °C for a total period of 72 h. After 24 h of cell culture initiation, cells were treated with different concentrations of PS-NPs (0–125 µg/mL). Two sets of duplicate cultures were used for each NPs concentration, two cultures were left untreated and served as untreated/negative control, and two cultures were treated with 45 µg/mL of doxorubicin (final concentration: 0.02 µg/mL) and were used as a positive control. To block cytokinesis and obtain binucleated cells, cytochalasin B (cytB) was added to the cell culture 44 h after cell culture initiation at a final concentration of 6 µg/mL, according to a delayed co-treatment protocol [37]. This protocol was used to reduce the possible effects of cytB on the cellular uptake of PS-NPs by endocytosis [37]. At 72 h of incubation, the cultures were harvested by centrifugation at 400× *g* for 10 min. After that, the cell pellet was treated with a cold hypotonic solution (0.0075 M KCl) to eliminate red blood cells and immediately centrifuged at 400× *g* for 10 min. Finally, cells were fixed three times with cold fixative (methanol: acetic acid, 5: 1; for the first fixation, fixative was diluted with an equal part of 0.9% NaCl), dropped onto clean, cold slides, and air-dried. Conventional staining with May-Grünwald/Giemsa stains was used. Slides were analyzed with a Nikon Eclipse E200 microscope. No less than a thousand cytochalasin B-blocked binucleated cells were analyzed per culture and concentration. The standard scoring criteria for MN were used [36]. Binucleated cells containing MN were documented using LUCIA Cytogenetics™ Database software (Laboratory Imaging, s.r.o., Prague, Czech Republic). To compare the proliferation status of the culture CBMN assay for lymphocyte samples treated with different nanoparticle concentrations, the cytokinesis-block proliferation index (CBPI) was calculated. The proportion of mononuclear (MONO), binucleated (BN), tri-, and tetranucleated (MULTIN) cells per 500 cells scored were calculated for each sample according to the equation [38]:

(2) $$CBPI=\frac{\left(1\times No.MONO\;cells\right)+\left(2No.BN\;cells\right)+\left(3No.MULTIN\;cells\right)}{\left(total\;number\;of\;cells\;scored\right)}$$

### 2.7. Statistical Analysis

Statistical methods were used according to the nature of the data and the type of analysis needed. Five independent cytotoxicity and comet assay experiments and three MN experiments were carried out. To assess the statistical significance of the obtained results, a Student’s *t*-test was applied. To describe the relationship between PS-NPs concentrations and induced primary DNA damage, as well as MN frequency, a linear regression model was evaluated. The findings were presented as mean ± SEM. *p* < 0.05 was considered as the level of significance.

## 3. Results

### 3.1. PS-NPs Characterization

**Figure 2:**
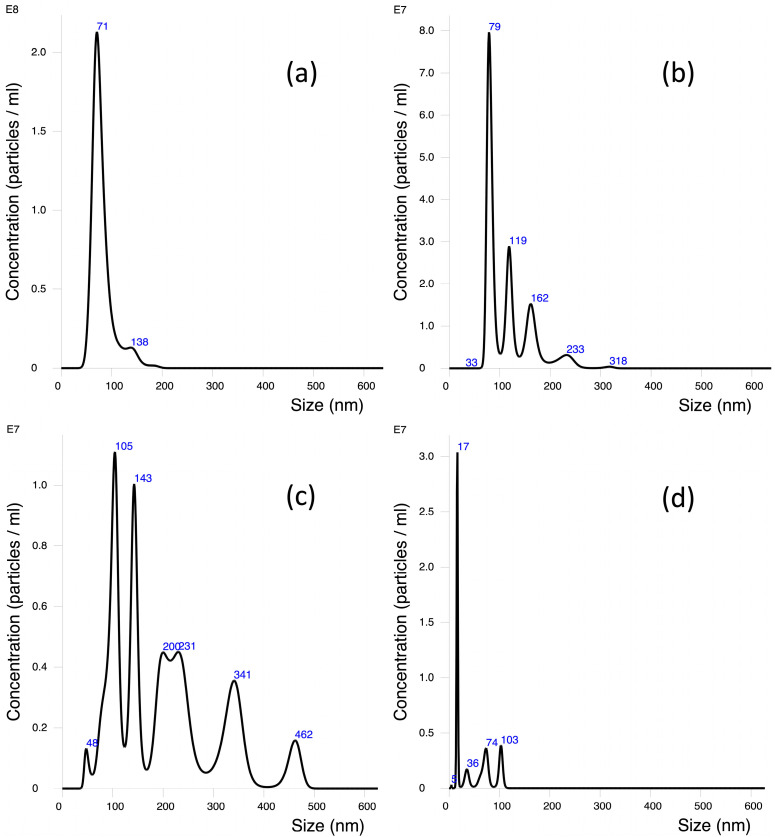
The hydrodynamic size distribution of polystyrene nanoparticles in water and RPMI 1640 cell culture media immediately after sonication (respectively, (**a**,**b**)) and in RPMI 1640 cell culture media 3 h (**c**) and 24 h (**d**) after sonication. Nanoparticles measured with NTA.

NTA analysis was performed to assess the hydrodynamic size of PS-NPs and levels of agglomeration in water (Figure 2a) and RPMI 1640 cell culture medium (Figure 2b–d).

The highest peak size (size distribution peak with most particles) and mean particle size distribution were determined by tracking analysis of Brownian motion immediately, 3 h, and 24 h after sonication (Table 1). The highest peak size of PS-NPs in water and RPMI 1640 medium immediately after sonication was similar (71 and 79 nm, respectively). However, the mean size was larger in RPMI 1640 cell culture medium sample. After 3 h of incubation of NPs in RPMI medium, mild agglomeration was determined, with the highest peak size of 105 nm and the mean size of 202 nm. Interestingly, after long-term incubation (24 h), levels of agglomeration were reduced. NTA analysis revealed that the mean size of the PS-NPs in the 24 h sample reached 47.5 nm, while the single distribution peak determined that most particles in this sample were 17 nm in size. Before every NTA measurement, samples were mixed very gently to avoid any mechanical impact on particles and possible agglomerates. The lower level of agglomeration and smaller size of NPs after a 24 h incubation in RPMI medium might be seen due to possible precipitation of larger agglomerates.

### 3.2. Uptake Analysis

**Figure 3:**
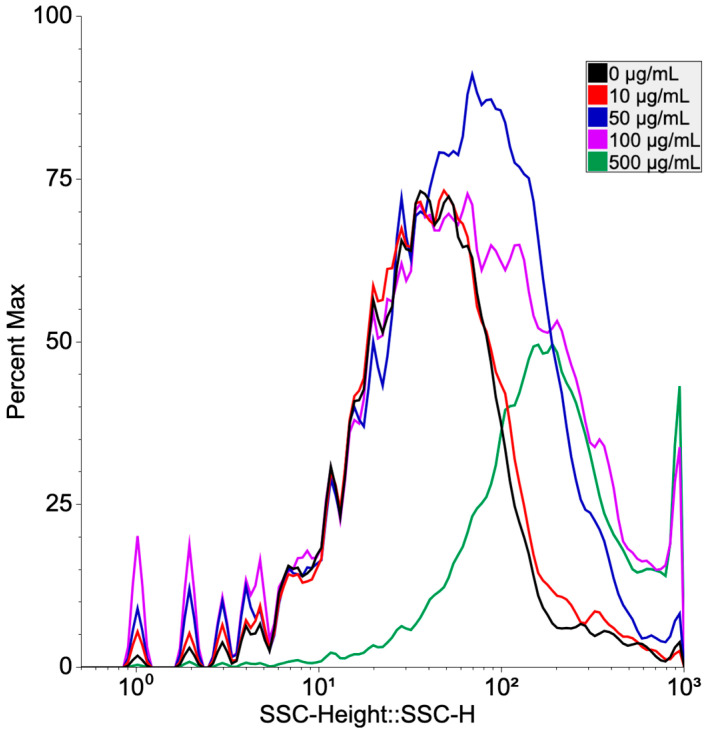
Analysis of incorporation of polystyrene nanoparticles by flow cytometric light scatter. Human PBMCs were treated with different concentrations (10, 50, 100, and 500 µg/mL) of PS-NPs for 24 h. SSC was evaluated using FCM.

To investigate the incorporation of PS-NPs by human PBMCs, flow cytometry side scattering (FCM SS) light analysis was applied. FCM graphs after 24 h exposure with different concentrations of PS-NPs (10–500 µg/mL) are shown in Figure 3. Yielded results showed that PS-NPs were incorporated into the cells in a concentration-dependent manner. The lowest side scattering light intensity was observed in untreated cells, while the highest SS intensity was observed in cells exposed to 100 and 500 µg/mL PS-NPs concentrations.

### 3.3. Cell Viability and Induction of Primary DNA Damage

**Figure 4:**
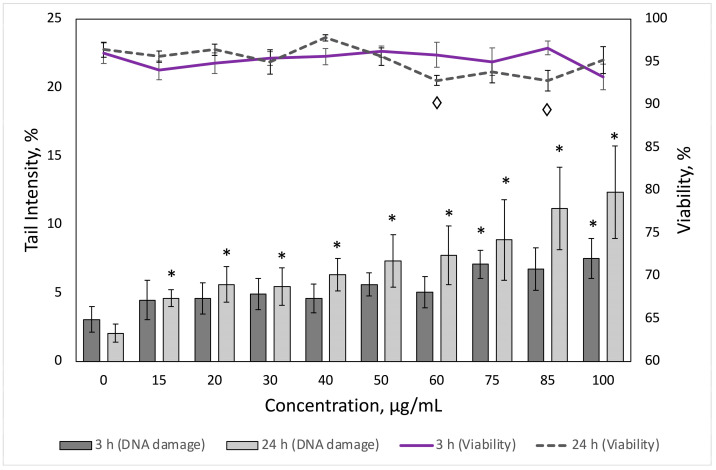
Results of primary DNA damage evaluation by comet assay (bar charts) and PBMC viability assessment using dual AO/EB staining technique (line charts). Percentage of viable cells and percentage of DNA in a comet tail after 3 h and 24 h exposure of human PBMCs to different concentrations of polystyrene nanoparticles. Results are presented as a mean value. A statistically significant increase (*p* < 0.05) as compared to the control cultures is indicated by an asterisk or rhumbs (Student’s *t*-test). Vertical bars indicate the standard error of the mean.

Cytotoxicity and primary DNA damage induced by PS-NPs were analyzed in PBMCs from five different donors. A dual AO/EB staining technique was performed to determine the cytotoxicity of tested PS-NPs concentrations (15–100 µg/mL) (Figure 4, line graph). Although a few concentrations statistically significantly reduced cell viability after 24 h incubation (60 and 85 µg/mL reduced cell viability to 92.8 ± 0.58% and 92.8 ± 1.2%, respectively), none of the tested PS-NPs concentrations reduced cell viability by more than 20%, regardless of incubation time, therefore, for the comet assay, concentrations of up to 100 µg/mL were used.

Induction of primary DNA damage, such as single-stranded breaks, double-stranded breaks, and apurinic and apyrimidinic sites, was evaluated to determine the genotoxic potential of PS-NPs using the alkaline comet assay. H_2_O_2_ treatment showed 3.3 and 5.8 times greater amounts of DNA damage at both time points (3 and 24 h, respectively) in comparison to untreated control. As shown in Figure 4 (bar charts), after 3 h of treatment, mild genotoxic effects were observed, 75 and 100 µg/mL concentrations induced a statistically significant increase in % TDNA, compared to untreated control (7.1 ± 1.03%, *p* = 0.01, and 7.53 ± 1.46%, *p* = 0.02, respectively). However, exposure to PS-NPs for 24 h substantially increased the amount of DNA damage. All tested PS-NPs concentrations induced significant amounts of DNA strand breaks. The most dramatic shift in primary DNA damage was observed at 85 and 100 µg/mL PS-NPs concentrations (respectively, 11.2 ± 3.02% and 12.35 ± 3.39%). At both time points (3 and 24 h), a very strong direct relationship between %TDNA and NP concentration was determined (R = 0.9447 and R = 0.9814). Dose-dependency could be described by equations %TDNA = 3.4606 + 0.0401 × C_NP_ and %TDNA = 2.7479 + 0.09267 × C_NP_, respectively, where %TDNA is the average percentage of DNA in the comet tail and C_NP_ is the concentration of PS-NPs. The relationship was found to be significant at both time points (R^2^ = 0.89, *p* < 0.001, R^2^ = 0.96, *p* < 0.001, respectively).

### 3.4. The Micronucleus Assay

**Figure 5:**
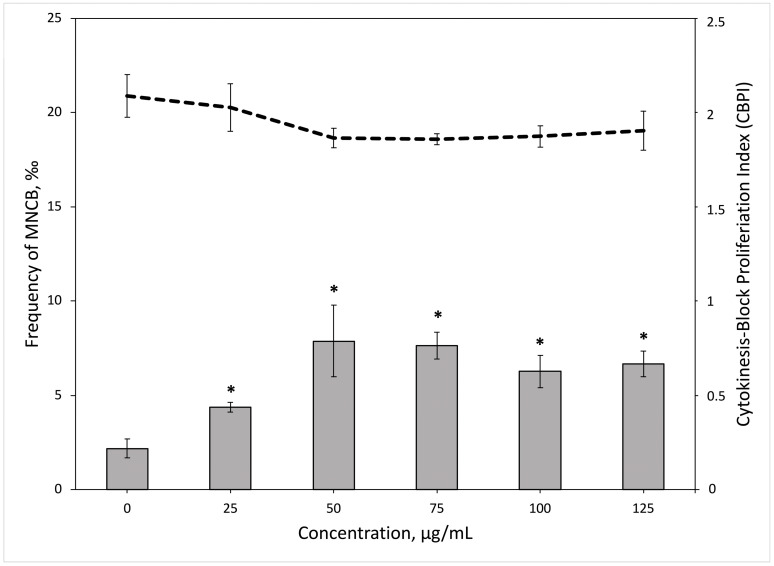
Frequency of micronucleated cytokinesis-blocked lymphocytes (bar charts) and cytokinesis-blocked proliferation index (line charts) in human peripheral blood lymphocytes exposed to polystyrene nanoparticles. Results are presented as mean values. Statistically significant differences (*p* < 0.05, Student’s *t*-test) from the untreated control cultures are indicated by an asterisk. Vertical bars indicate the standard error of the mean.

To evaluate the clastogenic and aneugenic potential of PS-NPs, the micronucleus assay was employed. Whole blood samples from three healthy donors were treated to PS-NPs concentrations up to 125 µg/mL, and the frequency of micronuclei and CBPI was assessed. Positive control showed a 7.8 times greater increase in the frequency of micronuclei in comparison to untreated control. As shown in Figure 5 (bar chart), the statistically significant frequency of micronuclei was induced in lymphocytes after the exposures with all tested PS-NPs concentrations (Student’s *t*-test, *p* = 0.02, *p* = 0.04, *p* = 0.003, *p* = 0.01, *p* = 0.006, respectively). However, no statistically significant dose–response relationship was found. All tested PS-NPs concentrations slightly affected cell proliferation, yet, differences were not statistically significant (Figure 5, dash line).

## 4. Discussion

The aim of this study was to assess the cytotoxicity and genotoxicity of polystyrene nanoparticles in human peripheral blood mononuclear cells. To assess the cyto-genotoxic potential of PS-NPs, we analyzed the uptake of these particles by human PBMCs and evaluated cell viability, DNA strand breaks, micronuclei formation, and CBPI. The analysis of flow cytometry side scattering light (FCM SS) intensity revealed that human PBMCs are able to internalize PS-NPs. PS-NPs concentrations up to 100 µg/mL were not cytotoxic to PBMCs, as evaluated with a dual AO/EB staining technique. However, yielded results showed that at all concentrations, the amount of DNA strand breaks gradually increased in a time and dose-dependent manner. In addition, tested PS-NPs concentrations were able to induce micronuclei formation without significant impact on cell proliferation.

The results of PS-NPs internalization analysis showed that these particles can be efficiently uptaken by human PBMCs after 24 h exposure and in a dose-dependent manner. As noticed by Rubio and colleagues [19] in a study with three lymphoblastic human cell lines (THP-1, TK6, Raji-B), extremely high internalization rates of polystyrene nanoparticles were observed after 24 and 48 h of exposure. At the highest concentration tested (50 µg/mL), nearly all of the cells contained PS-NPs. Similarly, a study with human white blood cells (WBCs) showed a high capacity of WBCs to uptake PS-NPs [23]. In this study, it was also proven by flow cytometry and confocal microscopy that the internalization rate of PS-NPs is cell type-specific. Ballesteros et al. observed that while lymphocytes did not show significant internalization levels, polymorphonuclear cells (PMNs) and monocytes exhibited a clear dose-dependent PS-NP uptake after 24 h of exposure [23]. Using coarse-grained molecular simulations, Rossi et al. [39] discovered that PS-NPs are able to penetrate lipid membranes. PS chains, when dissolved in the membrane core, change the structure of the membrane, drastically limit molecular transport, and soften the membrane. Furthermore, a study on nanoplastics and plasma proteins interactions [40] revealed that several plasma proteins display strong affinity to NPs and produce a multi-layered protein corona, resulting in more efficient cellular uptake and distribution of plastic nanoparticles.

Although we were able to confirm the uptake of PS-NPs by human PBMCs, we observed only mild cytotoxic effects; none of the tested PS-NPs concentrations (15–100 µg/mL) reduced cell viability by more than 20% at both time points (3 and 24 h). The selection of tested concentrations was based on a study that suggests that at 100 µg/mL concentration, PS-NPs induce a statistically significant increase in apoptotic cells [24]. Yet, another study showed that PS-NPs at higher concentrations of 500 µg/mL reduce lymphocyte viability to 73% [22]. As mentioned above, the uptake of PS-NPs by different cells was demonstrated in several studies [17,19,23,41], and interestingly, similar cytotoxicity results as in our study were obtained. No significant changes in white blood cell viability were found by Ballesteros et al. after 24, 48, and 72 h of exposure to PS-NPs (PP-008-10) [23]. Moreover, in a different study, it was shown that despite the PS-NPs’ size (29, 44, or 72 nm), concentrations up to 100 µg/mL were not cytotoxic to the human peripheral blood lymphocytes [24]. Similarly, such tendency was observed in leukocytic cell lines: Raji-B, TK6, and THP-1 treated with PS-NPs (PP-008-10) [19]. However, it was reported that higher PS-NPs concentrations (200–2000 µg/mL) can reduce blood cell viability significantly [19,22,24]. Yet, it is thought that effects found at extremely high concentrations may not be relevant from the environmental point of view [19].

Genotoxicity biomarkers can identify early consequences of the interaction between the individual and their environment. It is known that various xenobiotics can damage DNA and affect chromosome replication or gene transcription, which can result in cancer development or cell death [42]. Our research showed that after short- (3 h) and long-term (24 h) incubation, PS-NPs caused a concentration-dependent increase in DNA damage in PBMCs. As expected, longer incubation time produced a significantly higher amount of DNA damage (F = 6.91, *p* = 0.01). Compared to ordinary environmental contaminants, NPs may need more time to enter the cells and induce damage; therefore, 24 h or longer exposures are recommended for cyto-genotoxicity evaluation of nanoparticles [43]. After 24 h exposure, all tested NPs concentrations statistically significantly increased the level of DNA strand breaks, compared to untreated control. The highest amount of DNA damage was induced by the 85 and 100 µg/mL PS-NPs concentration (reaching 11.2 and 12.35% tail DNA, respectively). At both time points (3 and 24 h), DNA damage was induced in a dose-dependent manner. Experiments of similar design demonstrated that after 3 h of incubation PS-NPs concentrations (5–50 µg/mL) had only mild genotoxic effects on Raji-B and TK6 cells as evaluated by alkaline and FPG-modified comet assay [19]. Yet, after longer (24 and 48 h) treatment, PS-NPs induced a statistically significant increase in DNA damage. What is more, different genotoxicity mechanisms in TK6 and Raji-B cells were observed, where PS-NPs induced oxidative damage and likely direct DNA breaks, respectively [19]. The strong genotoxic effects of PS-NPs were demonstrated in monocytes and polymorphonuclear (PMN) cells treated with up to 100 µg/mL PS-NPs concentrations for 24 h. However, only mild genotoxic effects were observed in the lymphocytes [23].

Given that DNA damage detected by the comet assay could be repaired before it results in a mutation, additional genotoxicity biomarkers, such as induction of micronuclei, must be included [44]. In our study, all PS-NPs concentrations induced a statistically significant increase of MN, yet no direct relationship between these two markers was observed. Furthermore, a relationship between the reduction of CBPI and PS-NPs doses was observed. Previously, human fibroblast Hs27 cells were exposed to PS-NPs concentrations from 5 to 75 µg/mL, and results similar to the ones in our study were obtained. PS-NPs induced a statistically significant increase in MN frequency without any impact on the cell proliferation index [20]. Moreover, it was shown that pristine and surface-modified PS-NPs induced dose-dependent DNA damage in human A549 cells. Yet, surface-modified NPs were more genotoxic to A549 cells than unfunctionalized PS-NPs [21]. In a different study, a micronucleus test was conducted in human peripheral blood lymphocytes exposed to a range of PS-NPs concentrations (500–2000 µg/mL) [22]. The frequency of micronuclei increased significantly, and a clear dose–response relationship was observed. In addition, the nuclear division index decreased drastically compared to the untreated control. However, it was shown that tested concentrations affect cell hemolysis (increase up to 93%) and cell viability (reduction to 39%) as well, meaning PS-NPs might be rather cytotoxic and cytostatic at extremely high concentrations [22].

Compared to the alkaline comet assay results presented in this study, where all tested NP concentrations (15–100 µg/mL) induced a statistically significant increase in DNA damage after 24 h exposure in a dose-dependent manner, such a trend was not observed in the frequency of micronuclei. This may be due to the specificity of assays, where comet assay evaluates primary DNA damage and micronuclei test detects chromosomal damage. However, an interesting point was noted in a study by Ballesteros et al. [23], where the sensitivity of different white blood cells (lymphocytes, monocytes, and PMNs) to PS-NPs was assessed. It was observed that not only the uptake rate of PS-NPs by lymphocytes were limited, but tested concentrations did not induce a significant amount of DNA damage, while monocytes and PMNs were sensitive to PS-NP DNA damaging action. Supposing in the micronucleus assay, we only analyze lymphocytes (because of PHA in cell culture growth medium), while in the comet assay, where PHA is not used, it is possible that we have a mixture of WBCs, so the use of these two methods does not necessarily lead to identical results. However, this hypothesis should be tested further.

According to the findings of this investigation, tested polystyrene nanoparticles were effectively uptaken by human PBMCs, induced SSBs, and DSBs formation, and increased the frequency of micronuclei without significant impact on cell viability. Even though the majority of studies agree that PS-NPs concentrations up to 100 µg/mL may not have a cytotoxic effect but induce DNA damage to human blood cells, it is important to note that naturally occurring plastic particles might have a stronger effect. It was demonstrated that plastic particles tend to adsorb other environmental contaminants, including heavy metals [45], organic contaminants [46], and polycyclic aromatic hydrocarbons (PAHs) [47], which can heighten its cyto-genotoxic effects. Studies with commercially manufactured plastic particles are of great need to deepen the knowledge about the genotoxic potential of these particles in various cell lines and organisms. However, new methodologies and techniques are needed to be able to collect and analyze naturally occurring plastic NPs.

## Figures and Tables

**Table 1:** PS-NPs size distribution analysis in distilled H_2_O and RPMI culture medium at different time points after sonication by NTA analysis.

Matrix	Time after Sonication, h	Highest Peak Size, nm	Mean Size, nm (SD)
(a) Distilled H_2_O	0	71	80.4 (22.4)
(b) RPMI 1640	0	79	114.9 (45.9)
(c) RPMI 1640	3	105	202 (103.1)
(d) RPMI 1640	24	17	47.5 (34.1)

## Data Availability

Data generated during the study and included in this article will be available upon request to the corresponding author.
